# A socioeconomic related 'digital divide' exists in how, not if, young people use computers

**DOI:** 10.1371/journal.pone.0175011

**Published:** 2017-03-31

**Authors:** Courtenay Harris, Leon Straker, Clare Pollock

**Affiliations:** 1 School of Occupational Therapy and Social Work, Curtin University, Perth, Western Australia, Australia; 2 School of Physiotherapy, Curtin University, Perth, Western Australia, Australia; 3 Faculty of Health Sciences, Curtin University, Perth, Western Australia, Australia; Universita degli Studi di Perugia, ITALY

## Abstract

Government initiatives have tried to ensure uniform computer access for young people; however a divide related to socioeconomic status (SES) may still exist in the nature of information technology (IT) use. This study aimed to investigate this relationship in 1,351 Western Australian children between 6 and 17 years of age. All participants had computer access at school and 98.9% at home. Neighbourhood SES was related to computer use, IT activities, playing musical instruments, and participating in vigorous physical activity. Participants from higher SES neighbourhoods were more exposed to school computers, reading, playing musical instruments, and vigorous physical activity. Participants from lower SES neighbourhoods were more exposed to TV, electronic games, mobile phones, and non-academic computer activities at home. These patterns may impact future economic, academic, and health outcomes. Better insight into neighbourhood SES influences will assist in understanding and managing the impact of computer use on young people’s health and development.

## Introduction

Information technology (IT) is becoming an increasingly important part of young people’s daily lives across both school and home environments [1]. To illustrate, in 2011 in Australia the internet was used by 79% of school-aged children; with 92% of these children accessing from home and 86% from school [2]. In addition to IT being used by most young people, the use of IT by young people has been linked with academic [3–4] and health outcomes [5–6].

Globally access to computers has not been universal. This has created a ‘digital divide’ between those countries with high rates of access and those with low rates of access. Additionally, even within individual countries with high levels of computer access there is evidence of a ‘digital divide’ between different regions, areas, and neighbourhoods. For example, the Australian Bureau of Statistics (ABS) [2] found that households in major cities (75%) were more likely to have Internet access than their regional (64%) or remote (62%) counterparts. Computer and internet access have also been found to be related to socioeconomic status (SES) factors which cluster in neighbourhoods, such as family income, parental level of schooling, and parental job category [2–3,7].

Specifically regarding use of IT by young people, there is evidence of inequitable neighbourhood access to computers within both school and home environments. Access to computers at school is influenced by educational bureaucracies, politics, resource allocation, curriculum development, and school priorities. Students from highly resourced schools have significantly greater access to, and more frequent use of, computers and the internet than students from low resourced schools [8]. At home, young people who live in areas with higher SES factors (such as household income and parental education) were more likely to have been frequent, longer term users of IT than young people from low SES areas [3,5]. These disparities in access can be substantial, with evidence that home internet access can drop from 90% in households in the highest income quintile, to just 40% in households in the lowest income quintile [2].

Governments have recently responded to the inequities in neighbourhood access with policies to equalise school and home access to computers. For example, the Australian government has proposed to equip all secondary students from Years 9–12 with a computer and assist in funding high speed broadband internet services to 98% of all homes [9]. Similarly, in England the government has committed to all children having home computer and internet access for school and college work [10]. These governmental measures have been prompted by research which has highlighted the importance of the nature of computer use for young people’s health and development outcomes. For example, in regard to academic outcomes, Lei [4] found a negative association between entertainment/exploration technology use and student grade point average. In relation to musculoskeletal health and development, prolonged laptop and tablet computer use has been associated with young people reporting musculoskeletal discomfort [11]. Conversely, better school readiness and cognitive development have been observed in children who use a computer for 15–20 minutes every day than in those who do not [12]. Additionally, use of the internet [13] and the use of home computers for educational activities [4] have shown positive relationships with school academic performance.

Despite policy measures being put in place to assist with the inequities of computer *access*, the influence of neighbourhood may still be evident with IT use. The term ‘digital inequality’ was introduced to extend the digital divide concept to include access to different types of technology, as well as knowledge and skills in technology use, and the capacity of an individual to harness their skill [14]. In countries such as Australia, where there is minimal digital *access* divide for young people (with near universal school computer access and home computer access), digital inequality may still exist in the nature of computer use [15]. In this way, the digital divide concept may be more broadly understood as a divide in not only if IT is used (access) but also how it is used, as well as subsequent outcomes. How IT is used is likely to be influenced by a range of contextual factors [16] and a complex interplay between technological variables (e.g., access, use, and skill) and sociocultural variables (e.g., demographic, economic, and cultural) [17].

It is argued that the use of IT is embedded in a sociocultural context, such that young people arrive at school with varying access to, and experience with, different technologies [17]. As a result, the relationship between school performance and IT use may be a product of sociocultural context. However, current evidence for a relationship between neighbourhood and the nature of young people’s IT use is limited. Whilst some research has found no correlation between neighbourhood SES related factors and IT use [18], other studies have reported both positive and negative relationships [5,19–20]. The current study sought to explore evidence for a neighbourhood ‘digital divide’ in an environment with almost universal computer access. Specifically, the aim of this study was to investigate the association of neighbourhood with young people’s exposure to computers and other IT, including the amount and nature of IT use. A better understanding of this issue is seen as critical to developing appropriate policies to ensure adequate economic, academic, and health outcomes for young people.

## Methods

### Sample

The data described here came from a broader study exploring IT exposure and outcomes in young people. Pathway models linking exposure to musculoskeletal outcomes was explored in a previous publication [21], but this did not address the relationship between neighbourhood SES (NSES) and IT use. As previously published [21], 1,351 participants were recruited from school Years 1 to 11 at 10 local government, Catholic education, and independent private schools in Perth, Western Australia. The final sample comprised 792 boys and 559 girls across Year 1 (*n* = 150), Year 6 (*n* = 350), 9 (*n* = 350), and 11 (*n* = 350). Participating child numbers and mean (SD) ages are shown in Table 1.

**Table 1:** Sample demographics.

School year	Sample		Age (years)	
	Proposed	Actual	Mean	SD
**Year 1**	150	146	6.8	0.7
**Year 6**	350	350	11.3	1.0
**Year 9**	350	563	14.2	1.2
**Year 11**	350	292	16.3	1.2

Discrepancies between proposed and actual numbers were due to four Year 1 questionnaires not being returned, some young people being absent on the day of testing, Year 11 students having other commitments, students not adequately completing questionnaires, and one school requesting all Year 9 children be surveyed. Informed passive consent (via school principals) was gained prior to data collection (data in S1 Appendix). The Curtin University Human Research Ethics Committee approved the study.

### Survey tool

As described previously [21], the survey tool used was based on the Young People’s Activity Questionnaire (YAQ) [22] with additional questions around devices and activities. The general activities included: ‘watching TV shows and DVDs’, ‘writing and drawing with pens and pencils’, ‘reading books/ magazines’, ‘using mobile phone for calls and texts’, ‘playing a musical instrument’, ‘playing electronic games not on a computer’, and ‘vigorous physical activity’ (data in S2 Appendix). This tool explored IT exposure across individual, family, and neighbourhood settings and included a simplified version for parents of Year 1 students. The instrument covered demographic details (e.g., age, general activity levels), general computer anxiety [23] and computer flow [24], nature of computer activities (games, multimedia, letter/ story writing, learning programs, internet, email, chat room), height and weight, family variables (e.g., IT exposure and parental use), and neighbourhood variables (e.g., location and IT exposure). NSES was evaluated using the Index of Relative Socioeconomic Advantage Disadvantage (IRSAD) drawn from the 2001 national census data [25]. For more details about the instrument, see [21].

### Survey procedures

The parents of participants in Year 1 completed their questionnaire at home and returned the questionnaires to their teachers for collection. The researcher attended the class on a different day to collect the height and weight measurements. For all other participants the questionnaire was completed at school during class time with the researcher and relevant teacher present. For Year 6, 9 and 11 young people measurements were collected at the time of questionnaire completion.

### Data analysis

Descriptive statistics were used to describe the sample and Spearman rank correlation coefficients (r_s_) were used to examine the strength and direction of bivariate relationships between NSES and young people’s IT exposure and other activities. A three-step hierarchical multiple regression analysis was used to examine the relative influence of individual (age, gender, body mass index (BMI), computer related anxiety, flow, ability to have choice of computer activities, and physical activity); family (parental computer use, home computer bedroom use, and total number of home computers); and NSES variables on IT exposure and other activities. Models predicting home computer exposure used home flow and choice over use whereas models predicting school computer exposure used school flow and choice over computer use. Four groups of models examined: 1) amount of computer exposure, 2) nature of computer exposure, 3) amount of other IT exposure and 4) amount of other activity exposure. SPSS v15 was used for all analyses, with a critical alpha level of .05.

## Results

### Individual and family characteristics

41.3% of participants were female and 58.6% were male. All participants had access to computers and the internet at school. 98.9% of participants reported having access to a computer at home with 95.9% reporting internet/email access at home. 71.2% (962) of participants reported that both parents had used a computer, with 19.4% (262) reporting only one parent had used a computer and 8.3% (112) reported neither/ didn’t know if a parent had used a computer.

### NSES characteristics

The mean NSES of the sample population based on IRSAD was 1035.4 (94.0), with a range from 869.9 to 1207.3. Table 2 shows the distribution of IRSAD from the sample compared to the Western Australian and Australian IRSAD distributions [21].

**Table 2:** Distribution of index of relative social advantage and disadvantage for study sample, state and national populations.

Percentile	Australia	Western Australia	Sample
**Minimum**	597	602	869
**10%**	880	909	924
**25%**	928	932	929
**50%**	990	957	1034
**75%**	1068	1008	1117
**90%**	1141	1077	1165
**Maximum**	1313	1301	1207

Table 3 demonstrates the distribution of school and home computer and internet access across NSES deciles and the influence of the environment on the computer and internet access of participants. Computer and internet access in the home environment, although high, varied from 92.0%—100.0% across the NSES deciles. This is in contrast to universal (100.0%) computer and internet access at school. NSES was associated with home access to both computers (*x*^*2*^(9) = 17.04, *p* = .048) and to the internet (*x*^*2*^(9) = 21.33, *p* = .011).

**Table 3:** Percentage of participants with access to computers and internet at school and home by NSES decile.

NSES		School		Home	
Decile	Values	Computer	Internet	Computer	Internet
<10^th^	<924.5	100.0	100.0	96.8	92.0
10^th^–<20^th^	924.6–929.5	100.0	100.0	98.7	93.5
20^th^–<30^th^	929.6–973.5	100.0	100.0	100.0	93.4
30^th^–<40^th^	973.6–995	100.0	100.0	100.0	94.3
40^th^–<50^th^	995.1–1034.5	100.0	100.0	97.0	97.0
50^th^–<60^th^	1034.6–1062.2	100.0	100.0	100.0	97.1
60^th^–<70^th^	1062.3–1111.7	100.0	100.0	98.6	97.9
70^th^–<80^th^	1111.8–1129.7	100.0	100.0	100.0	100.0
80^th^–<90^th^	1129.8–1164.9	100.0	100.0	100.0	98.2
>90^th^	>1165	100.0	100.0	99.4	98.9
	**Mean**	**100.0%**	**100.0%**	**98.9%**	**95.9%**

*Note*. NSES = neighbourhood socioeconomic status.

### Influence of NSES

#### Amount of exposure

As shown in Table 4, in bivariate analysis, NSES was positively related to school computer weekly hours (r_s_ = .086, *p* = .004), monthly frequency (r_s_ = .148, *p* < .001) and longest durations (r_s_ = .088, *p* = .003), indicating that young people from an area of high advantage were more likely to use computers at school for more hours, more frequently and for longer durations. Conversely home computer use was negatively related with NSES for home computer weekly hours (r_s_ = -.157, *p* < .001), usual duration (r_s_ = -.220, *p* < .001) and longest duration (r_s_ = -.138, *p* < .001), indicating that young people from an area of disadvantage were more likely to use computers at home for more hours, more frequently and for longer durations.

**Table 4:** Associations of NSES and the amount of computer exposure: Correlation and 3 step prediction model statistics.

Outcome variables—computer exposure	Spearman’s rho correlation	Individual R squared change (Step 1)	Family R squared change (Step 2)	NSES R squared change Step 3)	Overall model R squared	Overall model F and p
**School**						
Weekly hours	.086**	.073**	.008*	.002	.083**	8.93, <.001
Monthly frequency	.148**	.077**	.004	.018**	.100**	11.27, <.001
Usual duration	-.033	.079**	.004	.001	.083**	9.07, <.001
Longest duration	.088**	.049**	.002	.005*	.055**	5.84, <.001
**Home**						
Weekly hours	-.157**	.199**	.045**	.011**	.255**	33.09, <.001
Monthly frequency	-.036	.268**	.011**	.000	.279**	39.07, <.001
Usual duration	-.220**	.198**	.027**	.029**	.254**	34.40, <.001
Longest duration	-.138**	.244**	.014**	.010**	.268**	36.89, <.001

Note.

* = p <.05,

** = p <.001.

NSES = neighbourhood socioeconomic status.

Table 4 also summarises the first group of models examining relationships between NSES and computer use. This further analysis demonstrates that even after taking into consideration a combination of individual and family variables a relationship between IT use and NSES is still evident, and NSES significantly predicted the *amount* of both school and home computer use. The R squared values show that the associations with home computer use were stronger than those with school computer use. In additional post hoc analysis, the 55 participants without internet access were excluded and the pattern of results remained the same.

#### Nature of exposure

Table 5 demonstrates positive bivariate correlations between NSES and the frequency of school computer activities such as playing games, learning games and emailing (.094 < r_s_ < .257, *p* < .001), indicating that young people from an area of high advantage were more likely to use these computer activities. The negative bivariate correlations between NSES and home computer activities such as multimedia, internet, email and chat rooms (-.056 < r_s_ < -.117, *p* < .044), indicate that young people from an area of disadvantage were more likely to use these home computer activities.

**Table 5:** Associations of NSES and the nature of school and home computer exposure: Correlation and 3 step prediction model statistics.

Outcome variables—computer exposure	Spearman’s rho correlation	Individual R squared change (Step 1)	Family R squared change (Step 2)	NSES R squared change (Step 3)	Overall model R squared	Overall model F and p
**School**						
Play games	.126**	.091**	.021**	.008**	.121**	13.95, <.001
Multimedia	-.010	.086**	.007*	.001	.094**	10.52, <.001
Write Letter	.029	.041**	.002	.000	.044**	4.63, <.001
Learning Programs	.094**	.040**	.006	.006*	.052**	5.60, <.001
Surf Internet	.033	.171**	.004	.001	.176**	21.69, <.001
Email	.257**	.064**	.015**	.050**	.129**	15.03, <.001
Chat rooms	.017	.070**	.009*	.654	.079**	8.70, <.001
**Home**						
Play games	.020	.225**	.008*	.001	.234**	30.80, <.001
Multimedia	-.079**	.166**	.010*	.003*	.179**	22.05, <.001
Write Letter	.009	.025**	.008*	.000	.032**	3.37, <.001
Learning Programs	.042	.012	.006	.000	.019*	1.90, =.035
Surf Internet	-.062*	.348**	.008*	.001	.356**	55.73, <.001
Email	-.056*	.238**	.010*	.001	.249**	33.43, <.001
Chat rooms	-.117**	.094**	.006	.011**	.110**	12.51, <.001

Note.

* = p <.05,

** = p <.001.

NSES = neighbourhood socioeconomic status.

In this paper’s second group of 3 step models, the combination of individual, family and NSES variables were used to predict the *nature* of school and computer use, as characterised by the frequency of participating in various computer activities (Table 5). Similar to the correlation analysis, after inclusion of individual and family variables, NSES still significantly predicted the frequency of a number of school and home computer activities including: gaming (school), learning programs (school), email (school), multimedia (home) and chat room (home) (.003 < change R squared < .050, *p* < .043) (Table 5). Additional post hoc analysis excluding the 55 participants without internet access resulted in the same pattern of NSES relationship, as for the analysis of the amount of computer use.

#### Other IT exposure

In bivariate analysis, as shown in Table 6, NSES was negatively correlated (-.060 < r_s_ < -.152, *p* < .038) with TV, mobile phones, and electronic games frequency, usual duration and longest duration (except longest duration of TV/DVD). This indicated that young people from an area of low advantage were more likely to use these media more frequently and for longer durations. The positive bivariate relationship between NSES and reading (.072 < r_s_ < .130, *p* < .008) indicates that young people from areas of advantage were more likely to be reading more frequently and for longer durations.

**Table 6:** Associations of NSES and other IT exposure: Correlation and 3 step prediction model statistics.

Outcome variables other IT types	Spearman’s rho correlation	Individual R squared change (Step 1)	Family R squared change (Step 2)	NSES R squared change Step 3)	Overall model R squared	Overall model F and p
**Reading**						
Monthly frequency	.130**	.111**	.010*	.009*	.130**	15.09, <.001
Usual duration	.072**	.027**	.008*	.004*	.038**	4.01, <.001
Longest duration	.087**	.035**	.009*	.004*	.048**	5.13, <.001
**Writing**						
Monthly frequency	.011	.033**	.008*	.000	.033**	4.33, <.001
Usual duration	-.048	.036**	.002	.001	.040**	4.12, <.001
Longest duration	.007	.024**	.002	.000	.026*	2.70, =.002
**Electronic game**						
Monthly frequency	-.060*	.200**	.000	.006*	.454**	26.20, <.001
Usual duration	-.100**	.156**	.003	.007*	.167**	14.10, <.001
Longest duration	-.069*	.176**	.001	.003	.180**	15.46, <.001
**TV/ DVD**						
Monthly frequency	-.092**	.004	.001	.004*	.008	.852, =.588
Usual duration	-.126**	.053**	.001	.007*	.062**	6.66, <.001
Longest duration	-.026	.064**	.004	.000	.068**	7.30, <.001
**Mobile phone**						
Monthly frequency	-.127**	.404**	.003	.005*	.412**	70.64, <.001
Duration	-.144**	.254**	.005*	.009**	.268**	36.85, <.001
Longest duration	-.152**	.199**	.007*	.014**	.221**	28.52, <.001

Note.

* = p <.05,

** = p <.001. NSES = neighbourhood socioeconomic status.

IT = information technology.

In this paper’s third group of 3-step models, after inclusion of individual and family variables NSES still significantly predicted the frequency of participants’ exposure to electronic games, reading and mobile phone use; the usual duration of participants’ exposure to electronic games, reading, TV/DVD and mobile phones; and the longest duration of reading and mobile phone use (Table 6). Again, post hoc analysis excluding the 55 participants without internet access resulted in the same pattern of NSES relationship.

### Influence of NSES on other activities

In bivariate analysis NSES was positively correlated with music activity participation frequency (r_s_ = .173, *p* < .001), usual duration (r_s_ = .143, *p* < .001) and longest duration (r_s_ = .144, *p* < .001) (see Table 7). Similarly, NSES was positively correlated with vigorous physical activity frequency (r_s_ = .068, *p* = .014) and longest duration (r_s_ = .058, p = .041), though not usual duration (r_s_ = .001, *p* = .982). This indicates that young people from areas of advantage are more likely to be participating in music and vigorous physical activities more frequently and for longer durations, as they were more likely to participate in reading.

**Table 7:** Associations of NSES and other activity exposure: Correlation and 3 step prediction model statistics.

Outcome variables—computerexposure	Spearman’s rho correlation	Individual R squared change (Step 1)	Family R squared change (Step 2)	NSES R squared change (Step 3)	Overall model R squared	Overall model F and p
**Playing Music**						
Monthly frequency	. 173**	.056**	.013*	.024**	.094**	10.10, <.001
Usual duration	.143**	.026**	.008*	.009*	.042**	4.27, <.001
Longest duration	.144**	.030**	.012*	.007*	.121**	5.05, <.001
**Vigorous physical activity**						
Monthly frequency	.068*	.033**	.007	.001	.032**	4.57, <.001
Usual duration	.001	.045**	.003	.000	.039**	5.04, <.001
Longest duration	.058*	.074**	.002	.002	.070**	8.63, <.001

Note.

* = p <.05,

** = p <.001.

NSES = neighbourhood socioeconomic status.

In the final group of 3 step models, after inclusion of individual and family variables NSES still significantly predicted frequency, usual duration and longest duration of participation in music (step three change R squared .007 < change R squared < .024 *p* < .001). After inclusion of individual and family variables there was a trend for NSES to significantly predict frequency and longest duration of vigorous physical activity (step 3 change R squared .000< change R squared < .002, .145< *p* < .894).

## Discussion

Given recent changes in the way digital technology divides are conceptualised, the purpose of this study was to explore whether inequalities in the nature of IT use still exist in an Australian sample with near universal access to IT. As predicted, this study demonstrated that young people’s access to computers at home and school was high across all neighbourhoods. Despite comparable computer access, there was still an association between neighbourhood and how the young people in this study used IT. The associations with how IT was being used indicate that the ‘digital divide’ does indeed extend beyond simple access. These findings add to the current literature which suggests the assumption that young people will be ‘digital natives’ and uniformly use the technology available to them is not valid [17, 26].

### High NSES

Young people from advantaged neighbourhoods (high NSES) were found to use school computers more frequently, for longer durations and for different tasks. It is interesting that a difference in exposure patterns within the school environment of this study sample existed at all. The current political agendas and resources to provide equal school computer access along with State curriculum guidelines on computer use, which cover all Western Australian schools, appear to have not been sufficient to prevent NSES related differences in computer use. Previous studies have found that school computer use is linked to improved educational attainment [3–4]. Therefore the increased use of school computers reported by high NSES young people is likely to provide an opportunity for extended educational attainment and improved academic skills. Similarly, the students from high NSES neighbourhoods were found to have higher frequencies of using computer learning programs. These education related uses further promote the development of academic skills. Additionally, these students were found to be more likely to read books and play musical instruments. As current literature shows the potential for increased academic skills with playing music [27], and with reading [28], the overall activity patterns of these students are likely to support enhanced academic skills.

Moderate to vigorous physical activity for at least 60 minutes per day for young people is recommended as part of government guidelines to promote optimum health for young people [29]. As students from high NSES neighbourhoods were found to participate in more vigorous physical activity, their lifestyles have the potential for increased health and physical development in addition to enhanced academic attainment.

Young people from high NSES neighbourhoods had more opportunity for developing their academic skills given the greater use of school computers, the type of computer activities performed, and the variety of other activities they participate in. It is therefore evident that the digital divide issue is not only to do with access to computers but also the nature of their use. For all young people to participate in activities that promote academic attainment and good health, the nature of computer tasks and the variety of other activities they participate in needs to be addressed when implementing evidence-based guidelines for wise use of computers [30]. Further action can be taken by schools on the assumption that, despite near equal access, the nature of young people’s technology use will differ and this is largely dependent on the interaction between the educational environment and the young person’s wider sociocultural context [17].

### Low NSES

This study showed that young people from disadvantaged neighbourhoods (low NSES) used home computers for greater durations and for different tasks. While an increased use of home computers could be viewed as compensatory for reduced school computer use, a closer examination of the nature of their home computer use indicated different issues. Young people from low NSES, were found to *not* be using home computers for learning programs and academic related activities, but for multimedia and chat room activities. Whilst multimedia may include the development of school based project work and presentations, it could also include such tasks as downloading video and music media. Coupled with the use of chat rooms for more social based home computer activities, young people from low NSES neighbourhoods appear to be more focused on multimedia and social computer use at home, rather than educationally focused activities. Increased social use of computers by young people from low NSES neighbourhoods was also reflected in the different types of IT used. These young people were found to have increased use of non-educational IT including TV / DVDs, mobile phones and electronic games. Higher levels of TV watching and computer gaming have been linked to decreased academic performance including reading, written expression and mathematics [5,31]. Therefore young people from low NSES / disadvantaged areas have a pattern of IT use which may not be conducive to academic enhancement. To bridge this gap, education settings have a responsibility to adapt and integrate educational technologies to better meet the diverse needs of this new generation of learners [17].

Increased home computer use by young people from neighbourhoods of low NSES was significant for all three duration measures used within this study, indicating that these students had greater mean weekly hours of use, usual duration and longest durations. Given that these young people are also using other recreational screen based media for long durations (e.g. modal daily use for lowest NSES decile were: TV usual duration 2–5 hours, home electronic game usual duration 1–2 hours, mobile phone usual duration 30–60 minutes), they are potentially accumulating significant exposure to sustained postures while using these various forms of IT. Sustained postures have been shown to relate to musculoskeletal discomfort with use of mobile phones, home computers, electronic games, and laptop/tablet computers by young people [11,32–33].

Additionally, it has been suggested that children’s screen time activities may reduce vigorous physical activity participation and contribute to increased childhood obesity rates [34]. As this study found young people from low NSES neighbourhoods participated in less vigorous physical activity and used computers for longer durations they may be at greater risk of poor health and physical development outcomes.

Suhyun and Jingyo [35] found a negative association between TV viewing time in adolescence and likelihood of completing high school or attaining a tertiary degree attainment. When viewing the patterns of IT related activity of young people from low NSES neighbourhoods in this study (reduced school computer use, longer durations of TV use, and socially orientated home computer use), it appears that these young people are more likely to *not* have the opportunity to develop their academic skills fully. This suggests that, even when issues concerning access to IT are addressed, the nature of IT use by young people in low NSES areas may be part of a vicious cycle of self-perpetuating SES disadvantage and have long lasting adverse consequences for economic, academic, and health outcomes.

### Limitations and strengths

Limitations of the study include the use of self-report for exposure estimates, lack of duration measures for specific school computer and home computer activities, lack of family based SES measures and the use of a cross-sectional study design. Child reports of family based SES measures such as parental education level and/or parental income and perceived socio-economic level were not used in this research, nor were government estimates of median income for the postcode of each participating school (e.g., [36]). Errors can occur with these methods as young people of different income families attend schools in different areas and young people may not fully understand their parent’s occupations, or know details of family income or parental education levels. This study used reports of young people’s primary resident postcode as it is expected that the young people were able to report their own suburb. Additionally, Turrell and colleagues [37] found that the use of local area postcode was adequate in measuring the nature and extent of mortality rates. Similarly, Olds and colleagues [38] used this method and found a relationship between NSES and screen time, however this was mainly due to TV viewing, not computer use or video game playing.

Given the importance of understanding the nature of IT use, multiple duration exposure measures of home computer and school computer activities would have assisted in a more complete understanding of the nature of computer use in these environments. Data collection was based on self-report for Years 6, 9, and 11. Self-report is known to increase error [39] compared with observation or direct measurement, but it is still the method of choice for exposure assessments in large samples (e.g. [40]). However, this method may have influenced the results by over- or under-estimating frequency, durations and the nature of IT use. This cross-sectional study was performed to gain an understanding of what was occurring across a range of ages and both genders at a point in time. Whilst this may not accurately demonstrate longitudinal patterns of IT use, it did allow a large sample of data to be collected at one time and captured a ‘snap shot’ of technology related activities which can be compared to later data as technology and usage patterns change rapidly [36]. Strengths of the study included a large, representative sample across a range of ages and including both genders and across a range of NSES, separate data on home and school IT use and on the nature of IT use, and a range of exposure measures.

### Conclusion

The findings from this study demonstrate that NSES was related to how young people used computers and participated in other activities. Therefore in a sample with near universal access to IT, issues of a digital divide can still be evident. NSES was clearly associated with the nature of young people’s current IT use and this may impact their future economic, academic, and health outcomes.

## Supporting information

S1 Appendix Parents information sheet. (PDF)

S2 Appendix Young people’s activity questionnaire. (PDF)

## Abbreviations

DVD: digital versatile disc
IRSAD: Index of Relative Socioeconomic Advantage Disadvantage
IT: Information technology
NSES: Neighbourhood Socioeconomic Status
SES: Socio economic status
TV: Television
VPA: Vigorous physical activity
