# Supplementary material for: A socioeconomic related 'digital divide' exists in how, not if, young people use computers
Source: PLoS One. 2017 Mar 31;12(3):e0175011. doi: 10.1371/journal.pone.0175011 (PMC5376329; doi:10.1371/journal.pone.0175011)
Supplement: S1 Appendix — (PDF) [file pone.0175011.s001.pdf]

KIDS AND COMPUTERS

Parent Information Sheet

Title of Study: Survey to Identify Risk Factors Associated with Children's Use of Information Technology (IT).

Chief Researcher: Ms Courtenay Harris, PhD student , 0417993269
School of Physiotherapy, Curtin University of Technology.

Purpose of the Study:
Children are participating in a range of activities everyday at school and home. Many of these activities involve the use of new technology such as desk top / laptop / palm computers, video games, electronic hand held games. These activities are engaging and research indicates that children are continuing to participate in these activities for longer durations and more frequently. Research to date on the effects of children participating in these types of activities indicate that some children experience discomfort as a result of their interaction.

This study will help us to better understand how children spend their time, the activities they participate in, and what activities and personal characteristics may contribute to children experiencing discomfort. In particular this study will help to identify factors effecting children with their use of both old and new Information Technology, eg. Reading and writing, computers, electronic games, watching television and playing video games.

This information will assist academics, educationalists and parents to understand the risk factors for school children's use of IT. This will further assist in making recommendations for wise use of IT by children. This will therefore enable and encourage children to use this valuable resource in educational, recreational and communication environments in a safe and productive manner.

Participants
This study involves surveying 1500 West Australian children from years one, six, nine and eleven during 2006. Surveying of Christian Brothers College Students will be occurring at the College during terms 3 and 4, with students from years nine and eleven participating in the research.

Procedures:
If you agree for your child to be in this study, you and / or your child will be asked to complete a questionnaire during school time. The questionnaire is approximately 16 pages and should take about 30 –40 minutes to complete. For students aged 5 or 6 years then you as the parent with your child will need to complete the questionnaire. If your child is aged 10 or more then it is expected that your child will complete the questionnaire themselves at school with their teacher and the chief researcher present. Your child's teacher will also answer a question regarding their current level in a subject.

When the questionnaires are completed they will be taken by the chief researcher to collate the data.

Risks, discomfort and benefits

Your child's participation in the study will only occur if you and your child voluntarily consent to participate. As your School Principal has given his consent for this study to take place during class time, the study will occur at school. If you DO NOT wish for your child to participate you MUST return the enclosed withdrawal form prior to completion of the questionnaires.

The costs to you and your child will be the time taken to complete the questionnaire, which we anticipate will be 30 – 40 minutes. There are no risks associated with participation in this research.

Your child will be expected to answer some questions related to their date of birth, suburb they live in, class teacher's name, initials, school they attend, and any history of health problems with their muscles, bones or joints. Questions will also be asked on what type of activities they participate in, eg. Writing, reading, drawing, playing on computers or electronic games, watching television, exercise and playing musical instruments.

The benefits of participating in this study is that you and your child will assist in the researcher understanding the effects of a range of activities on children at school and home. Information about the findings of this study and recommendations for safe use of IT by children will be made available to participants via the school they attend.

Approval will be gained from Curtin University of Technology Human Research Ethics Committee prior to commencement of this study.

Will my child's personal information be kept confidential?

The questionnaires are anonymous. All data will be stored in a locked room at the School of Physiotherapy. It will not be possible to identify your child or any other person participating in this study in any report on this research.

What if I want more information on the study?

If you require any further information regarding the study you are encouraged to contact the chief researcher or your child's school teacher and further clarification of your questions will be made available.

What if I want to Refuse or Withdraw my child from participating in the study?

Your child and yourself may refuse or withdraw from participating in the study at any time. If you choose to do this then your child will not be disadvantaged or prejudiced in any way, and arrangements will be made with the school to have your child participate in another activity during the time the other class members are completing the questionnaire. In the event that you withdraw you or your child needs to inform the chief researcher of this and all of your child's data will be destroyed.
