# Supplementary material for: A socioeconomic related 'digital divide' exists in how, not if, young people use computers
Source: PLoS One. 2017 Mar 31;12(3):e0175011. doi: 10.1371/journal.pone.0175011 (PMC5376329; doi:10.1371/journal.pone.0175011)
Supplement: S2 Appendix — (PDF) [file pone.0175011.s002.pdf]

Curtin University of Technology

Young People's Activity Questionnaire

YAQ

Many of the activities you do everyday at home and school affect you in different ways. They affect the way you think, move, act and play. The questions in this survey ask you about these activities and how you think they affect you. Your answers will help us to provide guidelines for these activities so that you can enjoy doing them.

There are three sections in this questionnaire
1. Questions asking general information about you
2. Questions about a range of different activities you do
3. Questions about using computers at school and home

Please fill in the boxes provided by either ticking the box, or writing your answer in the space available.

Thank you for answering these questions

Chief Researcher: **Ms Courtenay Harris**
Asscociate Professor: **Dr Leon Straker (School of Physiotherapy)**
Professor : **Dr Clare Pollock (School of Psychology)**

1. Questions about you and your school.

a. Which school do you go to?	☐
b. What year are you in at school?	☐ year/grade
c. Who is your class teacher?	☐
d. What suburb do you live in?	☐
e. When were you born?	☐ day ☐ month ☐ year
f. Are you a boy or girl?	1☐ boy 2☐ girl
g. Do you wear glasses or contact lenses?	1☐ yes 2☐ no
h. Which hand do you usually write with?	1☐ left 2☐ right 3☐ either

i. How many times in an average week do you do the following types of exercise for more that 15 minutes at a time?

**Hard** exercise that makes you puff and your heart beat fast – for example running, hockey, swimming training, dancing	☐
**Medium** exercise that is active but not exhausting – for example fast walking, gym, biking, skate boarding	☐
**Light** exercise that is not demanding – for example bowling, easy walking, playing or climbing outside or at the park	☐

j. When getting ready to go to school how often do you feel like you have butterflies in your stomach?

1☐ don't 2☐ 1 x month 3☐ 1 x week 4☐ 2-3 x week 5☐ daily

k. How much do you enjoy going to school?

1☐ really don't enjoy it 2☐ don't enjoy it 3☐ so so 4☐ like it 5☐ really like it

Now some questions about your health.

l. Have you <u>ever</u> had a problem with your muscles, bones or joints?

1❑ yes 2❑ no =► *if 'no' go to question p*

╚► *if* 'yes' *go to next question*

m. Please describe the problem with your muscles, bones or joints. (What it was [for example broken bone, scoliosis, arthritis], how long ago you had it, how it affects you now, why do you think ýou had it?)

....

....

n. In the <u>last month</u>, did you take any medicine to reduce the soreness you felt in your muscles, bones or joints?

1❑ yes 2❑ no

o. In the <u>last month</u>, did you see a doctor, physiotherapist, etc., because of the soreness you felt in your muscles, bones or joints?

1❑ yes 2❑ no

p. In the <u>last month</u> how often did you experience a headache?

1❑ not at all 2❑ 1 x month 3❑ 1 x week 4❑ 2-3 x week 5❑ daily

╚► *if 'not at all' go to question <u>'r'</u>*

q. In the <u>last month</u>, did you take any medicine to reduce the headache/s?

1❑ yes 2❑ no

r. In the <u>last month</u> how often did you experience any stomach pain, eg. nausea, vomiting, or cramps? (For girls this does not include period pain)

1❑ not at all 2❑ 1 x month 3❑ 1 x week 4❑ 2-3 x week 5❑ daily

╚► *if 'not at all' go to the next page*

s. In the <u>last month</u>, did you take any medicine to reduce the stomach pain/s?

1❑ yes 2❑ no

2. *Now some questions about **GENERAL ACTIVITIES** you do <u>each month</u>.*

In the <u>last month</u>, how often did you do the following activities? *Tick one box only for each activity.*

a. Watching TV shows and DVDs	1 ☐ not at all	2 ☐ 1 x month	3 ☐ 1 x week	4 ☐ 2-3 x week	5 ☐ daily
b. Writing and Drawing with pens and pencils	1 ☐ not at all	2 ☐ 1 x month	3 ☐ 1 x week	4 ☐ 2-3 x week	5 ☐ daily
c. Reading Books / Magazines	1 ☐ not at all	2 ☐ 1 x month	3 ☐ 1 x week	4 ☐ 2-3 x week	5 ☐ daily
d. Using a Mobile Phone for Calls or Texts	1 ☐ not at all	2 ☐ 1 x month	3 ☐ 1 x week	4 ☐ 2-3 x week	5 ☐ daily
e. Playing a Musical Instrument	1 ☐ not at all	2 ☐ 1 x month	3 ☐ 1 x week	4 ☐ 2-3 x week	5 ☐ daily

In the <u>last month</u>, for how long did you <u>usually</u> do the following activities each time? *Tick one box only for each activity.*

f. Watching TV shows and DVDs	1 ☐ never	2 ☐ < 30 minutes	3 ☐ 30-60 minutes	4 ☐ 1-2 hours	5 ☐ 2-5 hours	6 ☐ >5 hours
g. Writing and Drawing with pens and pencils	1 ☐ never	2 ☐ < 30 minutes	3 ☐ 30-60 minutes	4 ☐ 1-2 hours	5 ☐ 2-5 hours	6 ☐ >5 hours
h. Reading Books / Magazines	1 ☐ never	2 ☐ < 30 minutes	3 ☐ 30-60 minutes	4 ☐ 1-2 hours	5 ☐ 2-5 hours	6 ☐ >5 hours
i. Using a Mobile Phone for Calls or Texts	1 ☐ never	2 ☐ < 30 minutes	3 ☐ 30-60 minutes	4 ☐ 1-2 hours	5 ☐ 2-5 hours	6 ☐ >5 hours
j. Playing a Musical Instrument	1 ☐ never	2 ☐ < 30 minutes	3 ☐ 30-60 minutes	4 ☐ 1-2 hours	5 ☐ 2-5 hours	6 ☐ >5 hours

In the <u>last month</u>, what was the <u>longest time</u> you performed the following activities <u>without a break</u>? *Tick one box only for each activity.*

k. Watching TV shows and DVDs	1 ☐ never	2 ☐ < 30 minutes	3 ☐ 30-60 minutes	4 ☐ 1-2 hours	5 ☐ 2-5 hours	6 ☐ >5 hours
l. Writing and Drawing with pens and pencils	1 ☐ never	2 ☐ < 30 minutes	3 ☐ 30-60 minutes	4 ☐ 1-2 hours	5 ☐ 2-5 hours	6 ☐ >5 hours
m. Reading Books / Magazines	1 ☐ never	2 ☐ < 30 minutes	3 ☐ 30-60 minutes	4 ☐ 1-2 hours	5 ☐ 2-5 hours	6 ☐ >5 hours
n. Using a Mobile Phone for Calls or Texts	1 ☐ never	2 ☐ < 30 minutes	3 ☐ 30-60 minutes	4 ☐ 1-2 hours	5 ☐ 2-5 hours	6 ☐ >5 hours
o. Playing a Musical Instrument	1 ☐ never	2 ☐ < 30 minutes	3 ☐ 30-60 minutes	4 ☐ 1-2 hours	5 ☐ 2-5 hours	6 ☐ >5 hours

Now some final questions about GENERAL ACTIVITIES you do <u>each month</u>.

p. In the last month, how often did you feel any soreness in your muscles, bones or joints when you did any of these activities (**watch TV, read, write, use a mobile phone, play an instrument**?)

1☐ not at all 2☐ 1 x month 3☐ 1 x week 4☐ 2-3 x week 5☐ daily

└► *If* 'not at all' *go to question 3 on the next page* ═►

q. Which activity were you doing when you felt the soreness?

____

r. In the last month, did you ever stop the activity because of the soreness?

1☐ yes 2☐ no

s. In the last month, did you take any medicine to reduce the soreness you felt as a result of the activity?

1☐ yes 2☐ no

t. In the last month, did you see a doctor/physiotherapist/etc. because of the soreness you felt as a result of doing the activity?

1☐ yes 2☐ no

u. Circle each body part on the picture where you felt soreness while doing any of these general activities in the last month.

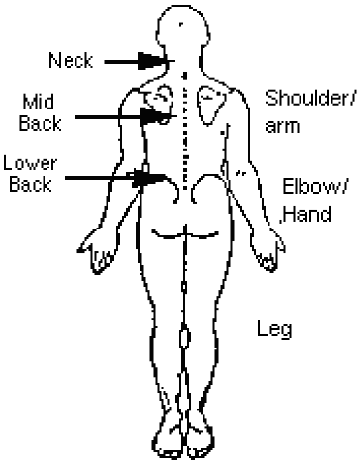

v. For each area you circled, rate how much soreness you had on a scale from 0 (no soreness) to 10 (extreme soreness).
Put the number from 0 to 10 in the box.

a neck ☐
b mid back ☐
(between your shoulder blades)
c lower back ☐
d left shoulder/arm ☐
e left elbow/hand ☐
f right shoulder/arm ☐
g right elbow/hand ☐
h legs ☐
I other …………… ☐

w. What do you think caused this soreness?

....

3. *Questions about* ***PLAYING ELECTRONIC GAMES.*** *(not on a computer)*

a. Do you have access to electronic games at home (not on a computer)?

1☐ yes 2☐ no

b. In the last month, how often did you play with electronic game devices? For example GameBoyTM, PSP, PlayStation, Xbox, hand held electronic games like 20Q, solitaire?

1☐ not at all 2☐ 1 x month 3☐ 1 x week 4☐ 2-3 x week 5☐ daily

► *If 'not at all' go to the next page* ═►

c. List the electronic game equipment you are most likely to use each month.

d. In the last month, for how long did you usually play electronic games each time?

1☐ < 30 minutes 2☐ 30-60 minutes 3☐ 1-2 hours 4☐ 2-5 hours 5☐ >5 hours

e. In the last month, what was the longest time you played electronic games without a break?

1☐ < 30 minutes 2☐ 30-60 minutes 3☐ 1-2 hours 4☐ 2-5 hours 5☐ >5 hours

f. In the last month, how often did you feel any soreness in your muscles, bones and joints when you played electronic games?

1☐ not at all 2☐ 1 x month 3☐ 1 x week 4☐ 2-3 x week 5☐ daily

► *If* 'not at all' *go to the next page* ═►

g. In the last month, did you ever stop playing electronic games because of the soreness?

1☐ yes 2☐ no

h. In the last month, did you take any medicine to reduce the soreness you felt as a result of playing electronic games?

1☐ yes 2☐ no

i. In the last month, did you see a doctor/physiotherapist/etc. because of the soreness you felt as a result of playing electronic games?

1☐ yes 2☐ no

j. Circle each body part on the picture where you felt soreness while playing electronic games in the last month.

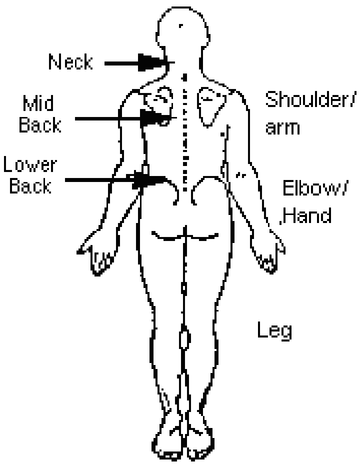

k. For each area you circled, rate how much soreness you had on a scale from 0 (no soreness) to 10 (extreme soreness). Put the number from 0 to 10 in the box.

a neck ☐
b mid back ☐ (between your shoulder blades)
c lower back ☐
d left shoulder/arm ☐
e left elbow/hand ☐
f right shoulder/arm ☐
g right elbow/hand ☐
h legs ☐
I other …………… ☐

l. What do you think caused this soreness?

....

4. Questions about *PHYSICAL ACTIVITIES like sport and exercise.*

a. In the last month, how often did you do vigorous physical activity (activities that make you puff or your heart beat faster like running, swimming, football, netball, hockey, dancing, bike riding, sport practice and games)?

1☐ not at all 2☐ 1 x month 3☐ 1 x week 4☐ 2-3 x week 5☐ daily

└► *If* 'not at all' *go to the next page* ═►

b. List the vigorous physical activities you most likely do each month.

c. In the last month, for how long did you usually do these physical activities each time?

1☐ < 30 minutes 2☐ 30-60 minutes 3☐ 1-2 hours 4☐ 2-5 hours 5☐ >5 hours

d. In the last month, what was the longest time you did these physical activities without a break?

1☐ < 30 minutes 2☐ 30-60 minutes 3☐ 1-2 hours 4☐ 2-5 hours 5☐ >5 hours

e. In the last month, how often did you feel any soreness in your muscles, bones and joints when you did these physical activities?

1☐ not at all 2☐ 1 x month 3☐ 1 x week 4☐ 2-3 x week 5☐ daily

└► *If* 'not at all' *go to the next page* ═►

f. In the last month, did you ever stop doing these physical activities because of the soreness?

1☐ yes 2☐ no

g. In the last month, did you take any medicine to reduce the soreness you felt as a result of doing these physical activities?

1☐ yes 2☐ no

h. In the last month, did you see a doctor/physiotherapist/etc. because of the soreness you felt as a result of doing these physical activities?

1☐ yes 2☐ no

i. Circle each body part on the picture where you felt soreness in the last month when doing these physical activities.

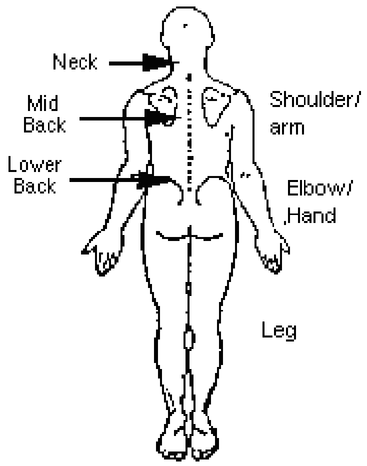

j. For each area you circled, rate how much soreness you had on a scale from 0 (no soreness) to 10 (extreme soreness). Put the number from 0 to 10 in the box.

a neck ☐
b mid back ☐ (between your shoulder blades)
c lower back ☐
d left shoulder/arm ☐
e left elbow/hand ☐
f right shoulder/arm ☐
g right elbow/hand ☐
h legs ☐
I other …………… ☐

k. What do you think caused this soreness?

....

5. *Questions about USING A DESKTOP OR LAPTOP COMPUTER*

a. Have you ever used a computer?

1☐ yes 2☐ no ═► *if* 'no' *go to question 6* ═►.

╚► *if* 'yes' *go to the next question*

b. About what age were you when you started using a computer?

☐ years old

The rest of this page is about using computers at ***SCHOOL only***

c. In the last month, how often did you use a computer at **school**?

1☐ not at all 2☐ 1 x month 3☐ 1 x week 4☐ 2-3 x week 5☐ daily

╚► *If* 'not at all' *go to question 6.* ═►

d. In the last month, for how long did you usually use a computer at **school** each time?

1☐ < 30 minutes 2☐ 30-60 minutes 3☐ 1-2 hours 4☐ 2-5 hours 5☐ >5 hours

e. In the last month, what was the longest time you used a computer at **school** without a break?

1☐ < 30 minutes 2☐ 30-60 minutes 3☐ 1-2 hours 4☐ 2-5 hours 5☐ >5 hours

f. In the last month, how often did you feel any soreness in your muscles, bones and joints when you used a computer at **school**?

1☐ not at all 2☐ 1 x month 3☐ 1 x week 4☐ 2-3 x week 5☐ daily

╚► *If* 'not at all' *go to the next page* ═►

g. In the last month, did you ever stop using a computer at **school** because of the soreness?

1☐ yes 2☐ no

h. In the last month, did you take any medicine to reduce the soreness you felt as a result of using a computer at **school**?

1☐ yes 2☐ no

i. In the last month, did you see a doctor/physiotherapist/etc. because of the soreness you felt as a result of using a computer at **school**?

1☐ yes 2☐ no

j. Circle each body part on the picture where you felt soreness in the last month when using a computer at school.

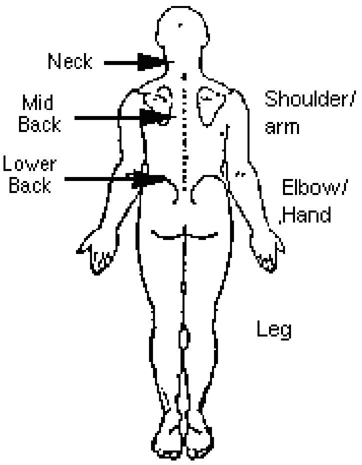

k. For each area you circled, rate how much soreness you had on a scale from 0 (no soreness) to 10 (extreme soreness). Put the number from 0 to 10 in the box.

a neck ☐
b mid back ☐ (between your shoulder blades)
c lower back ☐
d left shoulder/arm ☐
e left elbow/hand ☐
f right shoulder/arm ☐
g right elbow/hand ☐
h legs ☐
I other …………… ☐

l. What do you think caused this soreness?

....

Now some extra questions about your use of computers __AT SCHOOL.__

m. When using a computer at **school** which of these postures do you use? (tick all boxes that apply)

1❑ sitting at desk/table 2❑ sitting on floor 3❑ sitting on sofa/beanbag 4❑ lying down 5❑ standing 6❑ other

In the last month, how often did you do the following activities on a computer at **school**? (tick one box only for each activity)

n. Play games	1❑ not at all	2❑ 1 x month	3❑ 1 x week	4❑ 2-3 x week	5❑ daily
o. Use / create multi-media, eg pictures and music	1❑ not at all	2❑ 1 x month	3❑ 1 x week	4❑ 2-3 x week	5❑ daily
p. Write letters, stories etc.	1❑ not at all	2❑ 1 x month	3❑ 1 x week	4❑ 2-3 x week	5❑ daily
q. Use learning programs	1❑ not at all	2❑ 1 x month	3❑ 1 x week	4❑ 2-3 x week	5❑ daily
r. Surf the Net	1❑ not at all	2❑ 1 x month	3❑ 1 x week	4❑ 2-3 x week	5❑ daily
s. Send/receive emails	1❑ not at all	2❑ 1 x month	3❑ 1 x week	4❑ 2-3 x week	5❑ daily
t. Chat room	1❑ not at all	2❑ 1 x month	3❑ 1 x week	4❑ 2-3 x week	5❑ daily
u. Other activities	1❑ not at all	2❑ 1 x month	3❑ 1 x week	4❑ 2-3 x week	5❑ daily

v. In the last month, when using a computer at school how often did you use a computer in your classroom?

1❑ not at all 2❑ 1 x month 3❑ 1 x week 4❑ 2-3 x week 5❑ daily

w. In the last month, when using a computer at school how often did you use a computer in a computer lab?

1❑ not at all 2❑ 1 x month 3❑ 1 x week 4❑ 2-3 x week 5❑ daily

x. When you are using a computer at **school** who usually decides what you do?

1❑ me 2❑ friend 3❑ teacher 4❑ other person

y. How often can you choose what you do when using a computer at **school**?

1❑ always 2❑ usually 3❑ sometimes 4❑ rarely 5❑ never

z. Who else is usually with you when you are using a computer at **school**?

1❑ no one 2❑ friend 4❑ teacher 5❑ other

za. When using a computer at **school** who do you usually talk with?

1❑ no one 2❑ friend 4❑ teacher 5❑ other

zb. Thinking about the last seven days, in total how many hours have you spent using a computer at **school**? ▭ hours

zc. What type of computer do you use at school?

1❑ laptop 2❑ desktop 3❑ both desktop and laptop

Now the last questions about your use of computers AT SCHOOL.

Think about each time in the last week you used a computer at **school** while you are answering the following questions. (Tick one box per question ☑)

	strongly agree	moderately agree	slightly agree	don't agree or disagree	slightly disagree	moderately disagree	strongly disagree
zd. When using a computer at school, I felt I could make the computer do what I wanted it to.	1☐	2☐	3☐	4☐	5☐	6☐	7☐
ze. I felt the computer was more in charge of what happened than I was.	1☐	2☐	3☐	4☐	5☐	6☐	7☐
zf. When using a computer at school, I thought about other things.	1☐	2☐	3☐	4☐	5☐	6☐	7☐
zg. When using a computer at school, I noticed other things going on around me.	1☐	2☐	3☐	4☐	5☐	6☐	7☐
zh. When using a computer at school, I was totally absorbed in what I was doing.	1☐	2☐	3☐	4☐	5☐	6☐	7☐
zi. Using a computer at school excited my curiosity.	1☐	2☐	3☐	4☐	5☐	6☐	7☐
zj. Getting involved using a computer at school made me curious.	1☐	2☐	3☐	4☐	5☐	6☐	7☐
zk. Using a computer at school fired up my imagination.	1☐	2☐	3☐	4☐	5☐	6☐	7☐
zl. Using a computer at school bored me.	1☐	2☐	3☐	4☐	5☐	6☐	7☐
zm. Using a computer at school was interesting in itself.	1☐	2☐	3☐	4☐	5☐	6☐	7☐
zn. Using a computer at school was fun for me.	1☐	2☐	3☐	4☐	5☐	6☐	7☐

6. ***Questions about USING A COMPUTER AT <u>HOME ONLY</u>***

(this includes using a computer at a friend's home)

a. Do you have access to a computer at **home**?

1☐ yes 2☐ no

b. How many desktop computers do you have at **home**?

☐

c. How many laptop computers do you have at **home**?

☐

d. Do you have internet/email access at **home**?

1☐ yes 2☐ no

e. Does your mother/guardian use a computer?

1☐ yes 2☐ no 3☐ don't know

f. Does your father/guardian use a computer?

1☐ yes 2☐ no 3☐ don't know

g. In the <u>last month</u>, how often did you use a computer at **home**?

1☐ not at all 2☐ 1 x month 3☐ 1 x week 4☐ 2-3 x week 5☐ daily

└► *If* 'not at all' *go to question 7* ═►

h. In the <u>last month</u>, for how long did you <u>usually</u> use a computer at **home** each time?

1☐ < 30 minutes 2☐ 30-60 minutes 3☐ 1-2 hours 4☐ 2-5 hours 5☐ >5 hours

i. In the <u>last month</u>, what was the <u>longest time</u> you used a computer at **home** without a break?

1☐ < 30 minutes 2☐ 30-60 minutes 3☐ 1-2 hours 4☐ 2-5 hours 5☐ >5 hours

j. In the <u>last month</u>, how often did you feel any soreness in your muscles, bones and joints when you used a computer at **home**?

1☐ not at all 2☐ 1 x month 3☐ 1 x week 4☐ 2-3 x week 5☐ daily

└► *If* 'not at all' *go to q* ═►

k. In the <u>last month</u>, did you ever stop using a computer at **home** because of the soreness?

1☐ yes 2☐ no

l. In the <u>last month</u>, did you take any medicine to reduce the soreness you felt as a result of using a computer at **home**?

1☐ yes 2☐ no

m. In the <u>last month</u>, did you see a doctor/physiotherapist/etc. because of the soreness you felt as a result of using a computer at **home**?

1☐ yes 2☐ no

n. Circle each body part on the picture where you felt soreness in the last month when using a computer at home.

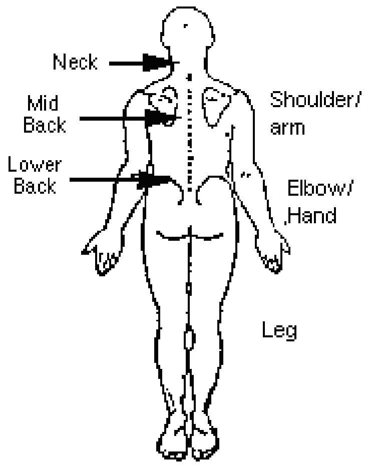

o. For each area you circled, rate how much soreness you had on a scale from 0 (no soreness) to 10 (extreme soreness). Put the number from 0 to 10 in the box.

- a neck ☐
- b mid back ☐ (between your shoulder blades)
- c lower back ☐
- d left shoulder/arm ☐
- e left elbow/hand ☐
- f right shoulder/arm ☐
- g right elbow/hand ☐
- h legs ☐
- I other …………… ☐

p. What do you think caused this soreness?
……

Now some extra questions about your use of computers ***AT HOME.***

q. When using a computer at **home**, which room are you usually in? (tick all boxes that apply)

1☐ shared area eg living room or shared study
2☐ your own bedroom or study
3☐ someone else's bedroom or study
4☐ some other room
5☐ varies, use it in different rooms

r. When using a computer at **home** which of these postures do you use? (tick all boxes that apply)

1☐ sitting at desk/table
2☐ sitting on floor
3☐ sitting on sofa/beanbag
4☐ lying down
5☐ standing
6☐ other

In the last month, how often did you do the following activities on a computer at **home**? (tick one box only for each activity)

s. Play games	1☐ not at all	2☐ 1 x month	3☐ 1 x week	4☐ 2-3 x week	5☐ daily
t. Use / create multimedia eg. pictures or music	1☐ not at all	2☐ 1 x month	3☐ 1 x week	4☐ 2-3 x week	5☐ daily
u. Write letters, stories etc.	1☐ not at all	2☐ 1 x month	3☐ 1 x week	4☐ 2-3 x week	5☐ daily
v. Use learning programs	1☐ not at all	2☐ 1 x month	3☐ 1 x week	4☐ 2-3 x week	5☐ daily
w. Surf the Net	1☐ not at all	2☐ 1 x month	3☐ 1 x week	4☐ 2-3 x week	5☐ daily
x. Send/receive emails	1☐ not at all	2☐ 1 x month	3☐ 1 x week	4☐ 2-3 x week	5☐ daily
y. Chat room	1☐ not at all	2☐ 1 x month	3☐ 1 x week	4☐ 2-3 x week	5☐ daily
z. Other activities, eg home work	1☐ not at all	2☐ 1 x month	3☐ 1 x week	4☐ 2-3 x week	5☐ daily

za. When you are using a computer at **home** who usually decides what you do?

1☐ me
2☐ friend
3☐ parent
4☐ other person

zb. How often can you choose what you do when using a computer at **home**?

1 always   2 usually   3 sometimes   4 rarely   5 never

zc. Who else is usually with you when you are using a computer at **home**?

1 no one   2 friend   3 brother/sister   4 parent   5 other

zd. When using a computer at **home** who do you usually talk with?

1 no one   2 friend   3 brother/sister   4 parent   5 other

ze. Thinking about the <u>last seven days</u>, in total how many hours have you spent using a computer at **home**? ☐ hours

zf. Tick the types of computers <u>you</u> use at **home**?

1 desktop   2 laptop   3 other

Now the last questions about your use of computers ***<u>AT HOME</u>.***

Think about each time in the last week you used a computer at **home** while you are answering the following questions. (Tick one box per question ☑)

	strongly agree	moderately agree	Slightly agree	don't agree or disagree	slightly disagree	moderately disagree	strongly disagree
zg. When using a computer at home, I felt I could make the computer do what I wanted it to.	1	2	3	4	5	6	7
zh. I felt the computer was more in charge of what happened than I was.	1	2	3	4	5	6	7
zi. When using a computer at home, I thought about other things.	1	2	3	4	5	6	7
zj. When using a computer at home, I noticed other things going on around me.	1	2	3	4	5	6	7
zk. When using a computer at home, I was totally absorbed in what I was doing.	1	2	3	4	5	6	7
zl. Using a computer at home excited my curiosity.	1	2	3	4	5	6	7
zm. Getting involved in a computer at home made me curious.	1	2	3	4	5	6	7
zn. Using a computer at home fired up my imagination.	1	2	3	4	5	6	7
zo. Using a computer at home bored me.	1	2	3	4	5	6	7
zp. Using a computer at home was interesting in itself.	1	2	3	4	5	6	7
zq. Using a computer at home was fun for me.	1	2	3	4	5	6	7

7. Finally, some questions about how you feel generally when you use computers ***anywhere.*** *(If you have never used a computer go to question 8)*

Please answer the next set of questions also by putting a tick in the box that best shows what you think.	strongly agree	moderately agree	Slightly agree	don't agree or disagree	slightly disagree	moderately disagree	strongly disagree
a. Computers do not scare me at all.	1☐	2☐	3☐	4☐	5☐	6☐	7☐
b. I do not feel anxious when other people talk about computers.	1☐	2☐	3☐	4☐	5☐	6☐	7☐
c. I get butterflies in the stomach when I think of trying to use a computer.	1☐	2☐	3☐	4☐	5☐	6☐	7☐
d. I would feel comfortable working with a computer.	1☐	2☐	3☐	4☐	5☐	6☐	7☐
e. Computers make me feel uneasy and confused.	1☐	2☐	3☐	4☐	5☐	6☐	7☐
f. I'm no good with computers.	1☐	2☐	3☐	4☐	5☐	6☐	7☐
g. Generally I would feel OK about trying a new problem on the computer.	1☐	2☐	3☐	4☐	5☐	6☐	7☐
h. I'm not the type to do well with computers.	1☐	2☐	3☐	4☐	5☐	6☐	7☐
i. I think using a computer would be very hard for me.	1☐	2☐	3☐	4☐	5☐	6☐	7☐
j. I have a lot of confidence in my ability when it comes to working with computers.	1☐	2☐	3☐	4☐	5☐	6☐	7☐

8. Before you finish this questionnaire please go to the researcher for height and weight measurements.

a. Height	cms
b. Weight	kgs

Thank you for your time and effort in completing this important questionnaire.
